# Comparison of Early vs. Delayed Anakinra Treatment in Patients With Adult Onset Still's Disease and Effect on Clinical and Laboratory Outcomes

**DOI:** 10.3389/fmed.2020.00042

**Published:** 2020-02-21

**Authors:** Antonio Vitale, Giulio Cavalli, Piero Ruscitti, Jurgen Sota, Serena Colafrancesco, Roberta Priori, Guido Valesini, Lorenza Maria Argolini, Elena Baldissera, Elena Bartoloni, Daniele Cammelli, Giovanni Canestrari, Elena Cavallaro, Maria Grazia Massaro, Paola Cipriani, Ginevra De Marchi, Salvatore De Vita, Giacomo Emmi, Micol Frassi, Roberto Gerli, Elisa Gremese, Florenzo Iannone, Marco Fornaro, Anna Paladini, Giuseppe Lopalco, Raffaele Manna, Alessandro Mathieu, Carlomaurizio Montecucco, Marta Mosca, Ilaria Piazza, Matteo Piga, Irene Pontikaki, Micol Romano, Silvia Rossi, Maurizio Rossini, Elena Silvestri, Chiara Stagnaro, Rosaria Talarico, Bruno Frediani, Angela Tincani, Ombretta Viapiana, Gianfranco Vitiello, Paola Galozzi, Paolo Sfriso, Carla Gaggiano, Salvatore Grosso, Donato Rigante, Lorenzo Dagna, Roberto Giacomelli, Luca Cantarini

**Affiliations:** ^1^Research Center of Systemic Autoinflammatory Diseases and Behçet's Disease Clinic, Department of Medical Sciences, Surgery and Neurosciences, University of Siena, Siena, Italy; ^2^Department of General and Specialized Medicine, Vita-Salute San Raffaele University, Milan, Italy; ^3^Unit of Immunology, Rheumatology, Allergy and Rare Diseases (UnIRAR), IRCCS San Raffaele Scientific Institute, Milan, Italy; ^4^Division of Rheumatology, Department of Biotechnological and Applied Clinical Science, University of L'Aquila, L'Aquila, Italy; ^5^Rheumatology Unit, Department of Internal Medicine and Medical Specialties, Sapienza University of Rome, Rome, Italy; ^6^Division of Rheumatology, ASST Gaetano Pini, Milan, Italy; ^7^Rheumatology Unit, Department of Medicine, University of Perugia, Perugia, Italy; ^8^Department of Experimental and Clinical Medicine, University of Florence, Florence, Italy; ^9^Institute of Rheumatology and Affine Sciences, Division of Rheumatology, Catholic University of the Sacred Heart, Rome, Italy; ^10^Department of Medical and Biological Sciences, Rheumatology Clinic, University of Udine, Udine, Italy; ^11^Periodic Fever Research Center, Institute of Internal Medicine, Catholic University of the Sacred Heart, Fondazione Policlinico A. Gemelli, Rome, Italy; ^12^Rheumatology and Clinical Immunology, Spedali Civili and Department of Clinical and Experimental Sciences, University of Brescia, Brescia, Italy; ^13^Rheumatology Unit, Department of Emergency and Organ Transplantation, University of Bari, Bari, Italy; ^14^Rheumatology Unit, Department of Medical Sciences, University and AOU of Cagliari, Cagliari, Italy; ^15^Department of Rheumatology, IRCCS Policlinico San Matteo Foundation, University of Pavia, Pavia, Italy; ^16^Rheumatology Unit, Department of Clinical and Experimental Medicine, University of Pisa, Pisa, Italy; ^17^Rheumatology Unit, Department of Medicine, University of Verona, Verona, Italy; ^18^Department of Medicine DIMED, Rheumatology Unit, University of Padua, Padua, Italy; ^19^Clinical Pediatrics, Department of Molecular Medicine and Development, University of Siena, Siena, Italy; ^20^Institute of Pediatrics, Università Cattolica Sacro Cuore, Fondazione Policlinico Universitario A. Gemelli IRCCS, Rome, Italy

**Keywords:** adult onset Still's disease, systemic onset juvenile idiopathic arthritis, autoinflammatory diseases, innovative biotechnologies, interleukin-1, anakinra, personalized medicine, treat to target

## Abstract

**Background:** Aim of this study was to search for any difference in the outcome of patients with adult onset Still's disease (AOSD) treated with anakinra (ANK) in relation with the interval between disease onset and the start of anti-interleukin(IL)-1 treatment and according with the different lines of ANK treatment.

**Patients and Methods:** One hundred and forty-one AOSD patients treated with ANK have been retrospectively assessed. Statistically significant differences (*p* < 0.05) were analyzed in the frequency of ANK effectiveness, primary or secondary inefficacy to ANK and rate of resolution of clinical and laboratory AOSD manifestations after 3, 6, and 12 months since ANK treatment according with different lines of treatment and different times between AOSD onset and start of ANK.

**Results:** No significant differences were identified in the ANK effectiveness and frequency of primary or secondary inefficacy for patients starting ANK within 6 months (*p* = 0.19, *p* = 0.14, and *p* = 0.81, respectively) or 12 months (*p* = 0.37, *p* = 0.23, and *p* = 0.81, respectively) since AOSD onset compared with patients starting ANK thereafter; no significant differences were identified in ANK effectiveness and primary or secondary inefficacy according with different lines of ANK treatment (*p* = 0.06, *p* = 0.19, and *p* = 0.13, respectively). Patients starting ANK within 6 and 12 months since AOSD onset showed a significantly quicker decrease of erythrocyte sedimentation rate and C-reactive protein than observed among patients undergoing ANK treatment after 6 and 12 months. The number of swollen joints at the 3 month follow-up visit was significantly lower among patients undergoing ANK within 6 months since AOSD onset (*p* = 0.01), while no significance was identified at the 6 and 12 month assessments (*p* = 0.23 and *p* = 0.45, respectively). At the 3 and 6 month visits, the number of swollen joints was significantly higher among patients previously treated with conventional and biological disease modifying anti-rheumatic drugs (DMARDs) compared with those formerly treated only with conventional DMARDs (*p* < 0.017).

**Conclusions:** Clinical and therapeutic outcomes are substantially independent of how early ANK treatment is started in AOSD patients. However, a faster ANK effectiveness in controlling systemic inflammation and resolving articular manifestations may be observed in patients benefiting from IL-1 inhibition as soon as after disease onset.

## Introduction

Adult onset Still's disease (AOSD) is a systemic multifactorial autoinflammatory disorder considered as the adult counterpart of systemic onset juvenile idiopathic arthritis (SOJIA) (1–6). According with the predominant symptoms, clinical presentation of AOSD can be classified into two main different patterns represented by a “systemic” type and a “chronic articular” type. Specifically, the systemic type includes patients mainly suffering from daily spiking fevers, typical salmon-like maculopapular rash, serositis, hepatosplenomegaly, and lymphadenopathy. Conversely, the chronic articular type includes patients mainly suffering from arthritis with less pronounced systemic inflammatory features. In turn, the systemic type can be distinguished into a monocyclic and polycyclic course, with the monocyclic form defined as a single flare lasting from 2 months to 1 year and the polycyclic type characterized by recurrent flares with asymptomatic intercritical periods (7).

Laboratory investigations typically show an elevated white blood cell count with neutrophil predominance, increased inflammatory markers, and high levels of serum ferritin. Serum liver enzymes are also increased in some patients (8). Currently, AOSD diagnosis is clinical and requires the exclusion of infectious, neoplastic and autoimmune diseases. Different sets of criteria have been developed for diagnostic and classification purposes, with Yamaguchi's criteria and Fautrel's criteria being the most frequently employed in the clinical practice (9, 10). To date, disease severity is assessed with the Pouchot's score modified by Pouchot et al. (8) and Rau et al. (11), which has also a prognostic impact and is useful in identifying patients at risk of AOSD-related death (12).

During the past years, non-steroidal anti-inflammatory drugs (NSAIDs) and corticosteroids represented the first-line treatment approach for AOSD patients, while conventional disease modifying anti-rheumatic drugs (cDMARDs), especially methotrexate, were used in resistant cases or as corticosteroid-sparing agents. Hydroxychloroquine and cyclosporine A represent additional cDMARDs usually employed in AOSD patients (13, 14). Among biotechnological agents, tumor necrosis factor (TNF)-α blockers have shown inadequate efficacy in controlling AOSD activity (15). On the other hand, interleukin-1 (IL-1) has proven to be a critical cytokine for the pathogenesis of both SOJIA and AOSD, thus inducing to include these disorders among multifactorial autoinflammatory diseases (16–19). On this basis and following the general improvement obtained in SOJIA patients, the IL-1 inhibitors anakinra (ANK) and canakinumab have been recently used in AOSD patients, leading to excellent clinical results even in cases resistant to conventional treatments (20–24). In particular, both clinical and laboratory manifestations resolve within a few days from the start of anti-IL-1 treatment in the majority of patients, also when a monotherapy approach is followed.

Despite the promising clinical results, not all patients are responsive to this treatment choice. Therefore, the identification of clinical or laboratory predictors of response currently accounts for a primary need to establish when and to whom IL-1 inhibitors should be started. In this regard, an early treatment with IL-1 blockade has been associated with a better outcome in the pediatric context (25–28).

The main aim of the present study is to identify any difference in the outcome of AOSD patients treated with ANK in relation with the interval between disease onset and start of the IL-1 inhibitor and according with the different lines of ANK treatment.

## Patients and Methods

### Patients and Data Collection

Patients included in this study have also been presented in other studies providing information about effectiveness of anti-IL-1 agents in AOSD patients, long-term drug retention rate of ANK, and predictive factors of response to ANK (24, 29).

Patients have been enrolled in 18 Italian tertiary Centers and had been diagnosed with AOSD basing on Yamaguchi's criteria (9). Before starting ANK, all patients had undergone a careful laboratory and radiologic screening in order to exclude infections, neoplasms and other rheumatologic disorders possibly inducing fever. Patients treated with ANK have been closely monitored with daily or weekly clinical and laboratory evaluations during the first month of treatment and then every three months or in case of either disease relapse or safety concerns.

A retrospective analysis of medical charts related to patients with AOSD was performed to evaluate any differences in the outcome of AOSD after treatment with ANK in relation with the time interval between disease onset and start of the IL-1 inhibitor and according with the different lines of ANK treatment. In particular, endpoints of the study were to identify any statistically significant difference in the frequency of global ANK effectiveness, primary or secondary inefficacy to ANK and in the resolution rate of clinical and laboratory AOSD manifestations after 3, 6 and 12 months of ANK treatment between: (i) patients starting treatment within 6 months since disease onset and patients undergoing treatment afterwards; (ii) patients starting ANK within 12 months since AOSD onset and patients undergoing treatment thereafter; (iii) patients treated with ANK before cDMARDs and/or other biologics; (iv) patients undergoing ANK after cDMARDs and before other biologics; (v) patients previously treated with both cDMARDs and other biologic agents. A further endpoint was to identify any statistically significant difference in the drug retention rate of ANK between patients treated with ANK before cDMARDs and other biologic agents, patients undergoing ANK after cDMARDs and before other biologics, and patients previously treated with both cDMARDs and other biologics.

The time cut-offs at 6 and 12 months after AOSD onset were chosen in order to accommodate the retrospective design of the study (patients were generally seen every three months in routine clinical practice) and the assessment of treatment outcomes based on how early IL-1 inhibition had been introduced.

Clinical variables considered in the statistical computation were: presence of fever, skin rash, pharyngodynia, arthritis, arthralgia, myalgia, pleuritis, pericarditis, liver involvement (hepatomegaly with increased liver enzymes), lymphadenitis (laterocervical and/or inguinal and/or axillary), lung involvement (non-infectious pneumonia), number of tender joints, number of swollen joints, disease activity score in 28 Joints-C-reactive protein (DAS28-CRP), baseline Pouchot score (hereinafter referred to as “systemic score”), decrease of systemic score at 3-month, 6-month and 12-month follow-up visits, daily corticosteroid dosage at the start of ANK, decrease in daily steroid dosage at 3-month, 6-month and 12-month follow-up visits.

Laboratory variables considered for the statistical computations were: total white blood cell counts, ferritin serum levels, erythrocyte sedimentation rate (ESR) and CRP.

The study protocol was conformed to the tenets of the Declaration of Helsinki and was approved by the local Ethics Committee of the University of Florence (reference number: 364-16OCT2013). Informed consent was obtained from each patient for the retrospective evaluation of her/his medical chart.

### Definitions

For diagnosis, fever was defined by a temperature higher than 39°C. At the 3-month, 6-month and 12-month follow-up visits patients were considered fever-free when body temperature was persistently lower than 37°C during the previous four weeks. Diagnosis of pleuritis, pericarditis and pneumonia was based on echographic-radiological documentation; hepatomegaly was documented by ultrasound, CT scan or magnetic resonance imaging (MRI). Regarding laboratory variables, white blood cell counts, ferritin, ESR and CRP were considered normal or increased according with the local laboratory normal ranges.

The concept of *effectiveness* was defined as “complete resolution or patient and physician's reported satisfactory resolution of clinical and laboratory AOSD manifestations”. A “*complete resolution of arthritis*” corresponded to the achievement of a DAS28-CRP <2.6, while the patient's reported satisfactory resolution corresponded to a patient global assessment ≤ 2.0 (scale 0–10) related to the overall disease activity and to the global health, separately; physician's reported satisfactory resolution corresponded to an evaluator global assessment ≤ 1.5 (scale 0–10) (30, 31). *Primary inefficacy* was considered as “no satisfactory improvement of clinical manifestations during the first four weeks of ANK treatment according with physician's judgment”. A *secondary inefficacy* was defined as “reappearance of AOSD manifestations for at least four weeks leading to ANK withdrawal despite a previous global response lasting at least 3 months”.

### Statistical Analysis

Descriptive statistics has included sample size, percentages, means, interquartile range (IQR), and standard deviations. After having assessed normality distribution with Shapiro-Wilk test, three-group comparisons of quantitative variables were performed by using ANOVA or Kruskall-Wallis test, as appropriate; qualitative variables were analyzed employing Chi-square test with 2x3 contingency tables. Unpaired two-tailed *t* test or Mann-Whitney two tailed U test with Bonferroni correction, as appropriate, were used for *post-hoc* analysis when global significance had been reached. Similarly, two-group comparisons were performed using two-tailed *t* test or Mann-Whitney two tailed U test for quantitative variables and by employing Chi-square test with 2x2 contingency tables for dichotomous data. When expected frequencies were less than five, Chi-square test was changed with Fisher exact test.

Drug retention rates (DRR) were assessed using the Kaplan-Meier plot, with “time 0” corresponding to the start of ANK treatment and the “event” being the discontinuation of therapy because of primary or secondary inefficacy. Log-rank (Mantel-Cox) test and Breslow test were used to compare differences in the initial and terminal part of different Kaplan-Meier curves, respectively.

In order to verify any role for the therapeutic interval between AOSD onset and the start of ANK and to search for confounding factors related to patients' features, AOSD activity and concomitant treatments, binary stepwise regression analysis was performed by using the following demographic, clinical and therapeutic features at the start of IL-1 inhibition as independent variables: disease duration (in months) between AOSD onset and the start of ANK, age at AOSD onset, AOSD type (systemic vs. chronic articular pattern), AOSD severity assessed with the systemic score, the daily corticosteroid dosage (mg/day of prednisone or equivalent), the concomitant use of cDMARDs, the number of tender joints, the number of swollen joints, the DAS28-CRP value, the sex of patients enrolled. The following outcomes were used as dependent variables: ANK effectiveness (established according to our definition) at 6- and 12-month follow-up visit; a systemic score equal to zero at 6 and 12 month follow-up visits; a DAS28-CRP <2.6 at 6- and 12-month assessments; the complete normalization of inflammatory markers (both ESR and CRP) at 6- and 12-month laboratory assessments.

Statistical Package for Social Science (SPSS) 24.0 package was used for statistical analysis. Two tailed *p*-values < 0.05 were considered statistically significant.

## Results

One hundred and forty-one patients (48 males, 93 females) diagnosed with AOSD according with Yamaguchi criteria and treated with ANK were enrolled in the study. Tables 1, 2 summarize demographic, clinical and therapeutic data of all patients recruited.

**Table 1:** General features of patients with AOSD at the start of treatment with anakinra.

**General AOSD features**	
Age at disease onset, years (mean ± SD)	35.3 ± 17.1
Age at diagnosis, years (mean ± SD)	37.32 ± 16.95
Disease duration before ANK treatment, months, mean (IQR)	50.4 (57)
Systemic disease pattern, *n* (%)	105 (74.5%)
Chronic articular pattern, *n* (%)	36 (25.5%)
**Clinical features**	
Number of tender joints, (mean ± SD)	6.6 ± 6.1
Number of swollen joints, (mean ± SD)	3.0 ± 4.2
DAS28-CRP, (mean ± SD)	4.5 ± 1.5
Pouchot (systemic) score, (mean ± SD)	5.58 ± 1.92
Fever, n (%)	136 (96.6)
Salmon-like skin rash, *n* (%)	104 (73.8)
Pharingodynia, *n* (%)	76 (53.9)
Arthritis, *n* (%)	99 (70.2)
Pleuritis, *n* (%)	21 (14.9)
Pericarditis, *n* (%)	26 (18.4)
Lymphadenitis, *n* (%)	73 (51.8)
Hepatomegaly, *n* (%)	66 (46.8)
Pneumonia, *n* (%)	10 (7.1)
**Increased laboratory markers, patients (%)**	
Erythrocyte sedimentation rate	120 (85.1)
C-reactive protein	129 (91.5)
Total white blood cells count	99 (70.2)
Serum ferritin	95 (67.4)
Liver enzymes	47 (33.3)

*ANK, Anakinra; AOSD, adult onset Still's disease; DAS28-CRP, disease activity score in 28 Joints-C-Reactive Protein; IQR, interquartile range; SD, standard deviation*.

**Table 2:** Treatment choices preceding and accompanying anakinra in the patients evaluated in our study.

**Treatments used before starting anakinra, n (%)**	
NSAIDs	97 (68.8)
Corticosteroids	138 (97.9)
cDMARDs	120 (85.1)
Methotrexate	91 (64.5)
Cyclosporine	50 (35.5)
Hydroxychloroquine	30 (21.3)
Colchicine	12 (8.5)
Azathioprine	9 (6.4)
Salazopyrine	8 (5.7)
Leflunomide	5 (3.5)
Gold salts	1 (0.7)
Intravenous immunoglobulins	1 (0.7)
Biotechnological agents	29 (20.6)
Etanercept	20 (14.9)
Infliximab	10 (7.1)
Adalimumab	6 (4.3)
Golimumab	2 (1.4)
Tocilizumab	2 (1.4)
Abatacept	2 (1.4)
Rituximab	2 (1.4)
Certolizumab	1 (0.7)
**Concomitant treatments at the start of anakinra, n (%)**	
cDMARDs	87 (61.7)
Methotrexate	63 (44.7)
Cyclosporine	15 (10.6)
Hydroxychloroquine	12 (8.5)
Colchicine	4 (2.8)
Leflunomide	2 (1.4)
Salazopyrine	2 (1.4)

*cDMARDs, conventional disease modifying anti-rheumatic drugs; n, number of cases; NSAIDs, non-steroidal anti-inflammatory drugs*.

Treatment with ANK was started in 40 patients (28.4%) within 6 months since AOSD onset and in 65 patients (46.1%) during the first 12 months of disease. In 19 cases (13.5%) ANK represented the first treatment approach soon after NSAIDs and corticosteroids; in 93 cases (66%) ANK treatment had been introduced as first-line biologic agent soon after cDMARDs failure, while other than anti-IL-1 biologic agents had been previously employed in 29 cases (20.6%). None of the patients had been treated with canakinumab before starting ANK.

The median disease duration at the start of ANK was: i) 2 (IQR = 3) months among patients starting ANK during the first 6 months of disease activity and 28.5 (IQR = 71.25) months among patients treated with ANK thereafter; ii) 5 (IQR = 7) months among patients starting ANK during the first 12 months from AOSD onset and 56 (IQR = 84) months among patients undergoing ANK afterward; iii) 3 (IQR=8) months among patients undergoing ANK as first treatment approach soon after NSAIDs and corticosteroids, 15 (IQR = 51) months among subjects treated with ANK after cDMARDs introduction and 69.5 (IQR = 164.75) months among patients undergoing ANK after cDMARDs and other biologics.

Dosages employed corresponded to ANK standard posology of 100 mg/day in 128 (90.8%) patients, while 4 (2.8%) patients were treated with higher dosages (200 mg/day), and 9 (6.4%) with lower than standard dosages (100 mg every other day or less).

### ANK Started Within 6 Months From Disease Onset

When analyzing differences in clinical outcome between patients starting treatment with ANK before and after 6 months of disease duration, no significant differences were identified in ANK effectiveness (*p* = 0.19) and in the frequency of primary (*p* = 0.14) or secondary (*p* = 0.81) inefficacy.

The systemic score at the start of treatments was significantly higher among patients undergoing ANK within 6 months since AOSD onset (6.3 ± 1.8 vs. 5.23 ± 1.9, *p* = 0.006). The decrease of systemic score at 3-, 6- and 12-month assessment was significantly higher among patients presenting a less than 6 months disease duration at the start of ANK (*p* = 0.006, *p* < 0.001 and *p* = 0.001, respectively). As reported in Table 3, the systemic score at 6-month assessment was significantly higher among patients starting ANK within six months from AOSD onset (*p* = 0.06), while no statistically significant differences were identified in the systemic score at 3- and 12-month assessments between the two groups (*p* = 0.67 and *p* = 0.89, respectively).

**Table 3:** Information about different responses in the articular manifestations between patients treated with anakinra no later than 6 months (upper part) and no later than 12 months (lower part) since AOSD onset compared with those starting the treatment thereafter.

	**Groups**	**Baseline**	***p*-value**	**At 3-months**	**p-value**	**At 6-months**	**p-value**	**At 12-months**	***p*-value**
Systemic score, mean ± SD	Group <6 months	6.3 ± 1.8	0.006	0.96 ± 1.0	0.67	0.64 ± 0.99	0.02	0.47 ± 1.07	0.89
	Group >6 months	5.2 ± 1.9		0.96 ± 1.4		0.52 ± 1.19		0.63 ± 1.21	
Tender joints, mean ± SD	Group <6 months	7.5 ± 6.6	0.81	1.9 ± 3.3	0.92	0.7 ± 1.4	0.33	1.8 ± 5.4	0.07
	Group >6 months	6.8 ± 5.9		2.0 ± 3.0		1.2 ± 3.1		0.5 ± 1.4	
Swollen joints, mean ± SD	Group <6 months	3.4 ± 4.3	0.63	0.4 ± 1.0	0.01	0.0 ± 0.0	0.23	0.4 ± 2.0	0.45
	Group >6 months	3.1 ± 4.4		0.6 ± 1.3		0.5 ± 1.6		0.2 ± 0.6	
DAS28-CRP, mean ± SD	Group <6 months	4.6 ± 1.7	0.50	1.8 ± 0.9	0.66	1.7 ± 1.0	0.65	1.6 ± 0.9	0.27
	Group >6 months	4.6 ± 1.4		2.5 ± 0.9		1.8 ± 0.8		1.9 ± 1.4	
Systemic score, mean ± SD	Group <12 months	6.2 ± 1.8	<0.001	1.00 ± 1.06	0.78	0.67 ± 1.1	0.22	0.57 ± 1.2	0.37
	Group >12 months	5.0 ± 1.8		0.93 ± 1.23		0.53 ± 1.01		0.48 ± 1.04	
Tender joints, mean ± SD	Group <12 months	5.3 ± 5.2	0.03	2.0 ± 3.2	0.94	0.9 ± 2.4	0.76	0.4 ± 0.9	0.14
	Group >12 months	7.8 ± 6.7		1.9 ± 2.8		1.1 ± 3.1		1.4 ± 4.5	
Swollen joints, mean ± SD	Group <12 months	2.1 ± 3.2	0.03	0.7 ± 1.5	0.09	0.4 ± 1.2	0.54	0.2 ± 0.6	0.73
	Group >12 months	3.8 ± 4.9		0.4 ± 1.0		0.3 ± 1.5		0.3 ± 1.5	
DAS28-CRP, mean ± SD	Group <12 months	4.3 ± 1.4	0.04	2.3 ± 1.2	0.75	1.8 ± 1.0	0.77	1.5 ± 0.9	0.14
	Group >12 months	4.8 ± 1.6		2.4 ± 1.1		1.9 ± 1.0		1.8 ± 1.2	

*DAS28-CRP, disease activity score in 28 Joints-C-Reactive Protein; SD, standard deviation*.

The number of swollen joints at the 3-month follow-up visit was significantly lower among patients undergoing ANK within 6 months since disease onset (*p* = 0.01), while no significant differences were identified at the start of ANK and at 6- and 12-month follow-up visits (*p* = 0.63, *p* = 0.23 and *p* = 0.45, respectively). No differences were identified in the number of tender joints and in the DAS28-CRP value among the two groups at the start of ANK (*p* = 0.81 and *p* = 0.50, respectively) and after 3 months (*p* = 0.91 and *p* = 0.66, respectively), 6 months (*p* = 0.47 and *p* = 0.65, respectively) and 12 months (*p* = 0.23 and *p*=0.27, respectively). Table 3 provides details about the different responses in joints between patients treated no later than 6 months since AOSD onset and those starting the treatment thereafter.

Baseline corticosteroid dosage was significantly higher among patients treated early with ANK (*p* < 0.0001). The decrease in the steroid dosage was not statistically significant between groups at the 3-month assessment (*p* = 0.064); conversely, it was significantly higher at both 6- and 12-month visits (*p* = 0.002 and *p* = 0.011, respectively) among patients starting ANK as early as 6 months since AOSD onset.

At the start of ANK, no differences were identified in the number of patients showing increased ESR (*p* = 0.86), while CRP was significantly more frequently increased among patients undergoing ANK within 6 months since AOSD onset (*p* = 0.023). At the 3-month evaluation ESR normalized in a significantly higher number of patients treated with ANK within the first 6 months since AOSD onset (*p* = 0.004). A similar trend was identified also for CRP without reaching a statistical significance (*p* = 0.054).

**Figure 1:**
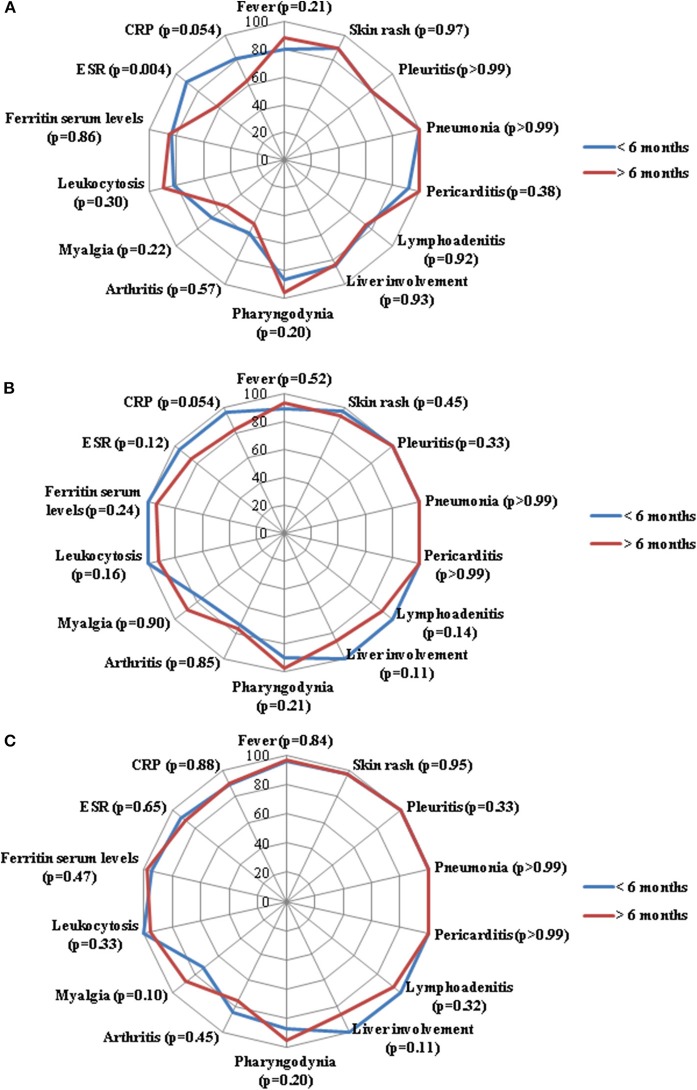
Radar graphics highlight the frequency of resolution of laboratory and clinical manifestations of adult onset Still's disease between patients starting anakinra (ANK) within the first 6 months since disease onset (<6 months) and those starting ANK thereafter (>6 months); **(A–C)** refer to the 3-month, 6-month and 12-month follow-up assessments, respectively. *P*-values were obtained by employing Chi square test. Alternatively, Fisher exact test was employed when expected frequencies were less than 5. CRP, C-reactive protein; ESR, erythrocyte sedimentation rate.

No statistically significant differences were identified in the frequency of serum ferritin normalization at the 3-month (*p* = 0.86), 6-month (*p* = 0.24) and 12-month (*p* = 0.47) assessments. No statistically significant differences were found between groups regarding leukocytosis at the 3-month (*p* = 0.30), 6-month (*p* = 0.16) and 12-month (*p* = 0.33) assessments. As detailed in Figure 1, no significant differences were identified in the frequency of resolution of specific clinical manifestations between the two groups of patients.

### ANK Started Within 12 Months From Disease Onset

No statistically significant differences were identified in the effectiveness of ANK (*p* = 0.37) and in the frequency of primary (*p* = 0.23) or secondary (*p* = 0.81) inefficacy between patients starting ANK during the first year since AOSD onset and those treated with ANK afterward.

At the start of treatment, systemic score was significantly higher among patients treated with ANK as soon as the first 12 months since the start of AOSD (6.2 ± 1.8 vs. 5.0 ± 1.8, *p* < 0.001). Similarly, the decrease of systemic score was significantly higher among patients treated with ANK as early as the first 12 months since disease onset at the 3-month visit (*p* = 0.012), 6-month visit (*p* = 0.001) and 12-month assessment (*p* = 0.002).

The baseline corticosteroid dosage was significantly higher among patients treated with ANK as soon as the first 12 months since AOSD onset (*p* = 0.001). Likewise, the decrease of steroid dosage was significantly higher among patients undergoing the earlier ANK treatment at the 3-month (*p* = 0.001), 6-month (*p* = 0.007) and 12-month (*p* = 0.032) follow-up visits.

Table 3 provides details about different responses in articular features between patients treated with ANK no later than 12 months since AOSD onset and those starting the treatment thereafter.

At the start of ANK no differences were identified in the number of patients showing increased ESR (*p* = 0.56) between the two groups of patients; conversely CRP was significantly more frequently increased among patients starting ANK during the first year of disease (*p* = 0.032). Among subjects treated with ANK during the first 12 months since AOSD onset, ESR normalized in a significantly higher number of patients at the 3-month assessment (*p* = 0.017), while no statistically significant differences were identified at the 6-month (*p* = 0.19) and 12-month (*p* = 0.58) follow-up visits. In the same group, CRP normalized in a significantly higher number of patients at the 3-month (*p* = 0.03) and 6-month (*p* = 0.014) visits, while no differences were found at the 12-month assessment (*p* = 0.83). No differences were disclosed in the frequency of leukocytosis resolution and serum ferritin normalization at the 3-month (*p* = 0.87 and *p* = 0.66, respectively), 6-month (*p* = 0.89 and *p* = 0.08, respectively) and 12-month (*p* = 0.92 and *p* = 0.13, respectively) assessments.

**Figure 2:**
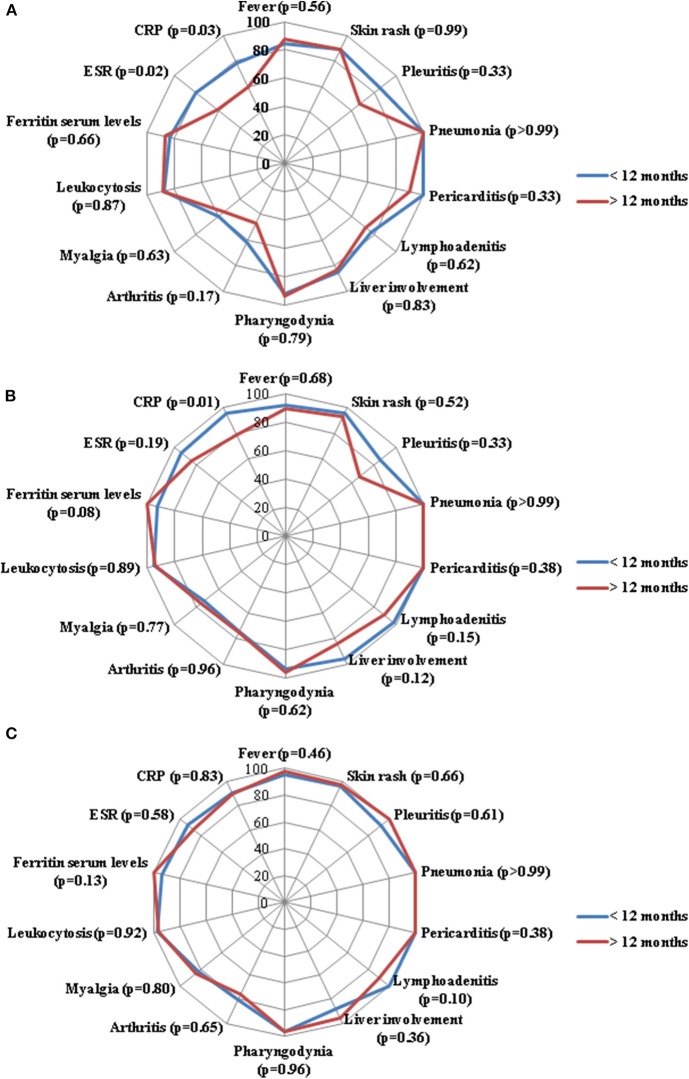
Radar graphics highlight the frequency of resolution of laboratory and clinical manifestations of adult onset Still's disease between patients starting anakinra (ANK) within the first 12 months since disease onset (<12 months) and those starting ANK thereafter (>12 months); **(A–C)** refer to the 3-month, 6-month and 12-month follow-up assessments, respectively. *P*-values were obtained by employing Chi square test. Alternatively, Fisher exact test was employed when expected frequencies were less than 5. CRP, C-reactive protein; ESR, erythrocyte sedimentation rate.

As shown in Figure 2, no significant differences were identified in the frequency of resolution of specific clinical AOSD manifestations between the two groups of patients.

### Different Lines of ANK Employment

No statistically significant differences were identified in ANK effectiveness (*p* = 0.06) and in frequency of primary (*p* = 0.19) or secondary (*p* = 0.13) inefficacy between patients treated with ANK after NSAIDs and corticosteroids, patients undergoing ANK as first-line biologic agent after cDMARDs failure and those previously treated with cDMARDs and other biologics. Table 4 describes clinical outcomes about systemic score, articular involvement and corticosteroid sparing effect between the three groups of patients.

**Table 4:** Differences about Pouchot score modified by Rau et al. (Systemic Score), articular involvement and corticosteroid sparing effect between patients undergoing anakinra (ANK) as first treatment approach and patients previously treated with conventional disease modifying anti-rheumatic drugs (cDMARDs) or with cDMARDs *plus* other biologic agents.

		**ANK first line** ** (group 1)**	**ANK preceded only by cDMARDs (group 2)**	**ANK preceded by cDMARDs and other biologics (group 3)**	***p*-value**
Systemic Score	Baseline	5.8 ± 1.4	5.5 ± 1.9	5.2 ± 1.0	0.17
Δ Systemic Score	3-month	5.3 ± 2.0 (91.4%)	4.6 ± 2.0 (83.6%)	3.3 ± 2.1 (63.5%)	**0.01**^**A**^
	6-month	5.4 ± 1.5 (93.1%)	4.9 ± 2.2 (89.1%)	4.5 ± 1.0 (86.5%)	**0.02**^**A**^
	12-month	5.7 ± 1.2 (98.3%)	4.9 ± 2.5 (89.1%)	5.0 ± 1.2 (96.2%)	**0.04**^**A**^
Tender joints	Baseline	6.9 ± 6.0	6.1 ± 4.9	4.5 ± 3.4	0.91
	3-month	0.8 ± 1.4 (88.4%)	2.1 ± 2.4 (65.5%)	2.0 ± 1.8 (55.5%)	0.50
	6-month	0.2 ± 0.7 (97.1%)	2.0 ± 3.1 (67.2%)	1.0 ± 1.4 (77.7%)	0.049*
	12-month	1.5 ± 3.9 (78.2%)	0.8 ± 3.1(86.8%)	0.5 ± 1.0 (88.8%)	0.85
Swollen joints	Baseline	3.8 ± 4.2	3.4 ± 4.5	3.5 ± 3.8	0.36
	3-month	0.3 ± 0.7 (92.1%)	0.4 ± 1.3 (88.2%)	2.0 ± 1.8 (42.8%)	**0.001**^**B**^
	6-month	0.0 ± 0.0 (100%)	0.2 ± 1.0 (94.1%)	0.8 ± 1.5 (77.1%)	**<0.0001**^**A, B**^
	12-month	0.7 ± 2.6 (81.5%)	0.1 ± 0.2 (97.0%)	0.5 ± 1.0 (85.7%)	0.08
DAS28-CRP	Baseline	5.0 ± 1.5	4.3 ± 1.5	3.4 ± 0.8	0.59
	3-month	1.9 ± 0.9 (62.0%)	2.4 ± 1.0 (44.1%)	2.4 ± 0.6 (29.4%)	0.22
	6-month	1.6 ± 0.8 (68.0%)	2.1 ± 0.9 (51.1%)	2.1 ± 0.6 (38.2%)	0.047*
	12-month	1.4 ± 0.6 (72.0%)	1.8 ± 0.8 (58.1%)	1.8 ± 0.6 (47.0%)	0.50
Corticosteroids, mg/day	Baseline	32.9 ± 14.4	16.8 ± 15.1	8.1 ± 3.8	**0.001**^**A, B**^
Δ Steroids, mg/day	3-month	22.1 ± 11.0 (67.2%)	7.9 ± 15.8 (47.0%)	0.6 ± 1.2 (7.4%)	**<0.0001**^**A, C**^
	6-month	29.2 ± 14.3 (88.8%)	11.7 ± 16.6 (69.6%)	3.3 ± 4.7 (40.8%)	**<0.0001**^**A, B**^
	12-month	31.1 ± 14.2 (94.5%)	13.8 ± 15.9 (82.1%)	5.1 ± 4.5 (63%)	**0.004**^**A**^

*Each bracketed percentage provides the reduction compared with baseline values*.

*DAS28-CRP, disease activity score in 28 Joints-C-Reactive Protein; the sign “Δ” indicates the mean decrease of Pouchot score or the daily corticosteroid dosage at the 3 follow-up visits compared with baseline*.

P-value were obtained by employing ANOVA or Kruskal-Wallis tests; for variables reaching global significance, post-hoc analysis was applied by using Mann-Whitney U test or Student t-test, as appropriate, with Bonferroni correction (p < 0.017). Significances at post-hoc analysis: A = group 1 vs. group 3; B = group 2 vs. group 3; C = group 1 vs. group 2. The sign “^*^” indicates the lack of significances at post-hoc analysis after Bonferroni correction (p>0.017). In the last column, the bold text was used to highlight p-values < 0.05.

**Figure 3:**
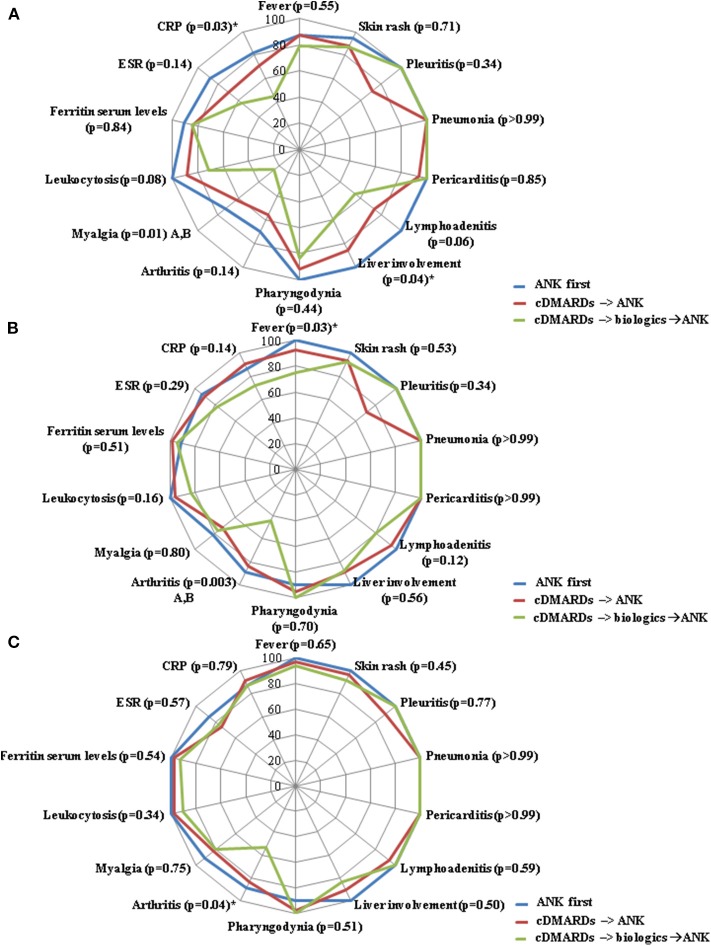
Radar graphics highlight the frequency of resolution of laboratory and clinical manifestations of adult onset Still's disease between: i) patients starting anakinra (ANK) before both conventional disease modifying anti-rheumatic drugs (cDMARDs) and other than anti-IL-1 biologic agents (*ANK first*); ii) patients treated with ANK after cDMARDs failure and before any other biologic agent (*cDMARDs* **→** *ANK*); and patients previously administered both cDMARDs and other biologics (*cDMARDs* **→** *biologics* **→** *ANK*). **(A–C)** refer to the 3-month, 6-month and 12-month follow-up assessments, respectively. *P*-values were obtained by employing Chi square test. Alternatively, Fisher exact test was employed when expected frequencies were less than 5. Significances at the *post-hoc* analysis: A = “*ANK first*” group vs. “*cDMARDs* **→** *biologics* **→** *ANK*” group; B = “*cDMARDs* **→** *ANK”* group vs. “*cDMARDs* **→** *biologics* **→** *ANK*” group. The sign “*” indicates a lack of significance at the Bonferroni correction (*p*>0.017).

No differences were identified in the serum ferritin levels between the three groups of patients at the 3-month, 6-month, and 12-month assessments. Figure 3 describes the frequency of resolution of laboratory and clinical AOSD manifestations at the 3-month, 6-month and 12-month follow-up visits. Table 5 provides details about the clinical manifestations at the start of ANK among patients starting treatment before and after 6 months since AOSD onset, before and after 12 months since AOSD onset and according with different lines of ANK employment.

**Table 5:** Clinical and laboratory manifestations at the start of anakinra (ANK) treatment among patients undergoing ANK within and after 6 months since adult onset Still's disease (AOSD) onset, patients starting ANK within and after 12 months since AOSD onset, patients treated with ANK before both conventional disease modifying anti-rheumatic drugs (cDMARDs) and biologics (*ANK first*), patients treated with ANK after cDMARDs failure and before any other biologic agent (*cDMARDs*→*ANK*), and patients previously administered both cDMARDs and other biologics (*cDMARDs*→*biologics*→*ANK*).

**Clinical manifestations**	**Delay** **<6 months (40 patients)**	**Delay** **> 6 months (101patients)**	***p*-value**	**Delay** **<12 months (65 patients)**	**Delay > 12** **months (76 patients)**	***p*-value**	**ANK first (19 patients)**	**cDMARDs** **→ ANK** **(93 patients)**	**cDMARDs →** **biologics → ANK (27 patients)**	***p*-value**
Fever	39 (97.5)	97 (96)	0.67	62 (95.4)	74 (97.4)	0.53	18 (94.7)	89 (95.7)	27 (100)	0.64
Skin rash	37 (92.5)	67 (66.3)	0.001	56 (86.2)	48 (63.2)	0.002	18 (94.7)	69 (74.2)	15 (55.6)	0.0002^A^
Pleuritis	11 (27.5)	10 (10)	0.008	15 (23.1)	6 (7.9)	0.012	4 (21.1)	14 (15.1)	3 (11.1)	0.63
Pneumonia	3 (7.5)	7 (6.9)	0.91	5 (7.7)	5 (6.6)	0.80	4 (21.1)	4 (4.3)	2 (7.4)	0.03*
Pericarditis	12 (30)	14 (13.9)	0.026	13 (20)	13 (17.1)	0.66	5 (26.3)	18 (19.4)	3 (11.1)	0.38
Lymphadenitis	24 (60)	49 (48.5)	0.22	42 (64.6)	31 (40.8)	0.005	13 (68.4)	46 (49.5)	12 (44.4)	0.24
Liver involvement	20 (50)	46 (45.5)	0.63	34 (52.3)	32 (43.8)	0.23	11 (57.9)	44 (47.3)	10 (37)	0.59
Pharyngodynia	25 (62.5)	51 (50.5)	0.20	42 (64.6)	34 (42.1)	0.018	12 (63.2)	53 (57)	9 (33.3)	0.06
Arthritis	26 (65)	73 (72.3)	0.40	44 (67.7)	55 (72.4)	0.58	11 (57.9)	64 (68.8)	23 (85.2)	0.11
Myalgia	34 (85)	71 (70.3)	0.07	51 (78.5)	54 (71.1)	0.32	14 (73.7)	72 (77.4)	18 (66.7)	0.52
Leukocytosis	31 (77.5)	68 (67.3)	0.23	52 (80)	47 (61.8)	0.019	15 (78.9)	66 (71)	16 (59.3)	0.33
Increased ferritin serum levels	30 (75)	66 (65.3)	0.27	49 (75.4)	47 (61.8)	0.08	14 (73.7)	61 (65.6)	20 (74.1)	0.61
Increased ESR	34 (85)	87 (86.1)	0.86	57 (87.7)	64 (84.2)	0.56	19 (100)	78 (83.9)	22 (81.5)	0.11
Increased CRP	40 (100)	89 (88.1)	0.023	63 (96.9)	66 (86.8)	0.032	19 (100)	86 (92.5)	22 (81.5)	0.07

*Values are patient numbers and, in brackets, percentages referring to each patient group. CRP, C-reactive protein; ESR, erythrocyte sedimentation rate*.

P-values were obtained by employing Chi square test. Alternatively, Fisher exact test was employed when expected frequencies were less than 5. Significances at the post-hoc analysis: A = “ANK first” group vs. “cDMARDs → biologics → ANK” group. The sign “*” indicates a lack of significance at the Bonferroni correction (p > 0.017).

Supplementary Table 1 provides details about the frequency of effectiveness to anakinra, primary and secondary inefficacy in the different subgroups of patients identified in the study. Similarly, supplementary Tables 2, 3 respectively add information about the mean values of the systemic score [according to Rau et al. (11)] and the mean daily corticosteroid dosage at 3-, 6- and 12-month assessments in the different subgroups of patients identified in the study. The mean decrease of the systemic score and of the daily corticosteroid dosage compared to the start of ANK are also reported.

### Drug Retention Rates Analysis

**Figure 4:**
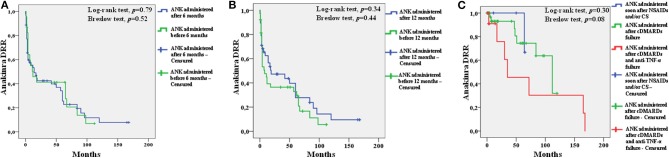
Kaplan-Meier survival curves of anakinra (ANK) obtained by distinguishing: i) patients starting ANK during the first 6 months since onset of adult onset Still's disease (AOSD) and patients starting ANK thereafter **(A)**; ii) patients starting ANK during the first 12 months since AOSD onset and patients starting ANK afterward **(B)**; patients starting ANK before both conventional disease modifying anti-rheumatic drugs (cDMARDs) and biologics (soon after non steroidal anti-inflammatory drugs and/or corticosteroids, *blue line*) from patients treated with ANK after cDMARDs failure (*green line*) and patients previously administered both cDMARDs and other biologics (*red line*) **(C)**. In order to focus attention to ANK efficacy, survival analysis excluded patients discontinuing ANK because of adverse events (25 cases), long-term disease remission (20 patients), lack/loss of compliance or other non-medical reasons (19 patients). One patient was also excluded owing to the lack of information about the overall treatment duration.

Figure 4 shows the different drug retention rates of ANK obtained including patients discontinuing treatment because of primary or secondary inefficacy.

After excluding patients who discontinued ANK because of adverse events, long-term disease remission, lack/loss of compliance or other non-medical reasons and 1 patient with no information about the overall treatment duration, no differences were identified in the drug retention rate of ANK between: (i) 23 patients treated with ANK as soon as the first 6 months since the start of AOSD manifestations and 53 patients starting ANK afterward (Log-rank test, *p* = 0.79; Breslow test, *p* = 0.52); (ii) 38 patients treated with ANK as soon as the first 12 months since the start of AOSD manifestations and 38 patients starting ANK afterward (Log-rank test, *p* = 0.34; Breslow test, *p* = 0.44); (iii) 10 patients treated with ANK as first treatment approach soon after NSAIDs and/or corticosteroids, 49 patients treated with ANK soon after cDMARDs failure and 15 patients treated with ANK after cDMARDs and other biologics failure (Log-rank test, *p* = 0.30; Breslow test, *p* = 0.08).

The median follow-up while on ANK treatment duration was: 4 (IQR = 34) months among patients undergoing ANK within 6 months from AOSD onset and 48 (IQR = 67) months among patients starting the treatment thereafter; 15.5 (IQR = 57) months among patients treated with ANK within 12 months from disease onset and 5.5 (IQR = 56) months in the group of patients starting the IL-1 inhibitor afterward; 7.5 (IQR = 62) months among patients treated with ANK as first treatment line, 10.5 (IQR = 55) months among patients treated with ANK after cDMARDs failure and 4 (IQR = 33) months among patients starting ANK after cDMARDs and other biologics.

### Regression Analysis

At binary stepwise regression analysis the therapeutic delay between AOSD onset and the start of ANK along with the age at AOSD onset, the AOSD pattern (systemic vs. chronic articular), the baseline systemic score, the daily dosage of corticosteroids, the concomitant use of cDMARDs, the number of tender joints, the number of swollen joints and the DAS28-CRP at the start of ANK did not predict the therapeutic outcomes consisting of: ANK effectiveness at 6- and 12-month follow-up visit; a systemic Pouchot score of zero at 6 and 12 month assessments; DAS28-CRP <2.6 at 6- and 12-month assessments; complete resolution of both ESR and CRP at 6- and 12-month visits. Supplementary Table 4 provides *p*-values obtained at the regression analysis.

## Discussion

During the last decade, the inhibition of IL-1 has been proven to be an effective treatment choice in different autoinflammatory disorders (6, 29, 32) and also in the majority of patients with AOSD, in whom a complete clinical and laboratory control of inflammatory manifestations is reached within a few days since the start of IL-1 blockers. Furthermore, anti-IL-1 therapy is associated with a significant corticosteroid sparing effect and a reduced need of concomitant cDMARDs. As a whole, such drug-sparing effect leads to a significant reduction of side effects, contributing to the improvement of patients' compliance, prognosis and long-term outcome (20–24, 33).

Based on different clues mainly reported in pediatric patients, some authors have suggested that early use of anti-IL-1 agents should be also considered for the initial treatment of SOJIA in order to reduce or avoid long-term complications, including severe joint damage and corticosteroid dependency. In this regard, pediatric patients could benefit from a “window of opportunity”, namely as a period of time after the disease onset during which the inhibition of IL-1 may effectively alter the course of disease and maybe induce disease remission or even disease resolution (25–28, 34, 35). In this regard, we conducted the present study to identify any effect on clinical and laboratory outcomes in relation with the time delay between disease onset and the start of the IL-1 inhibitor. Similarly, we searched for any difference in the therapeutic outcome of ANK administered in different timepoints of disease course with respect to other treatment choices.

According with our results, no significant differences are recognized in both the overall ANK effectiveness and frequency of primary or secondary inefficacy on the basis of the different intervals analyzed (6 and 12 months) as well as according to the different treatment lines. Similarly, the time delay between AOSD onset and the start of ANK did not represent a predictor of treatment outcome considered as ANK effectiveness, complete AOSD control according to the systemic score, achievement of a DAS28-CRP <2.6 and complete resolution of inflammatory markers.

On the other hand, a higher decrease of the systemic score and a more evident corticosteroid sparing effect were observed among patients treated earlier with ANK and in patients undergoing ANK as a first-line treatment approach. This statistical significances may be partially addressed to a more aggressive therapeutic decision making of physicians in the more severe cases, as revealed by the significantly higher baseline systemic score and daily corticosteroid dosage among patients treated with ANK within 6 and 12 months from disease onset compared with patients starting the treatment thereafter. Nevertheless, while no statistical differences were observed in the baseline systemic score according to different treatment lines (Table 4), the decrease of the score was significantly higher at 3-, 6-, and 12-month assessments among patients undergoing ANK as first treatment line compared to subjects previously treated with both cDMARDs and other biologics. This could be related to a more resistant inflammatory condition among patients experiencing several treatment failures, but it could also suggest a higher ANK effectiveness in controlling systemic disease activity when used early.

Conversely, the significantly higher systemic score identified at 6-month visit among patients starting ANK within the first six months from AOSD onset is hardly clinically significant, given the absence of statistical significances at both the previous and at the next time point, as well as in relation to the very modest mean values assumed by the score in the two groups (0.64 vs. 0.52).

Notably, patients starting ANK within six or twelve months since AOSD onset showed a quicker reduction of the inflammatory markers ERS and CRP than observed among patients undergoing ANK treatment after six and twelve months, respectively. This finding is even more pronounced when the 12-month time point is considered. However, statistical significance in the rate of ESR and CRP resolution between groups tends to disappear over time. In detail, while increased inflammatory markers were significantly more frequent at the 3-month assessment among patients starting ANK after both 6 and 12 months of disease duration, at the 6-month assessment the significance was solely limited to CRP, which was found more frequently increased among patients starting ANK after one year of disease duration. Lastly, no differences among groups were observed at the 12-month assessment. As a whole, these results suggest a slower activity of ANK in controlling systemic inflammation when employed later.

A similar trend was observed in the ability of ANK to control articular involvement. This is especially interesting, as joint affection has been frequently found less responsive to IL-1 inhibition than other AOSD manifestations (22, 23). Moreover, we recently found that the risk for a loss of efficacy of ANK increases along with the number of swollen joints at the start of treatment (29).

In the present study, the number of swollen joints at the 3-month follow-up visit was significantly higher among patients starting ANK after 6 months of AOSD duration compared with patients starting the biologic agent earlier. Conversely, no statistically significant differences were observed in the number of swollen joints at the 6- and 12-month follow-up visits. On the other hand, no statistical significances were observed about the number of tender joints and the DAS28-CRP at the three follow-up visits, neither considering the 6-month nor the 12-month time points.

Of note, the rate of arthritis resolution did not show statistical differences between patients starting ANK before and after 6 months or 12 months since AOSD onset. Conversely, as observed in Table 4, at the 3- and 6-month assessment the number of swollen joints was significantly lower among patients starting ANK soon after cDMARDs than those previously treated with cDMARDs and other biologics. In addition, at the 6-month visit the number of swollen joints was significantly lower among patients starting ANK as first-line treatment than among subjects treated with cDMARDs first. This finding seems to reconnect with a previous experience in SOJIA patients supporting a better treatment outcome among patients with no previous anti-TNF-α medications (27). In this regard, since a higher number of treatment lines generally reflects a longer disease history, the results obtained in our study seem to highlight a faster resolution of articular involvement in patients undergoing ANK during the first months of treatment.

The DRR analysis did not disclose statistically significant differences between patients treated with ANK during the first 6 or 12 months of AOSD duration compared to those undergoing IL-1 inhibition thereafter. Similarly, no differences were observed between patients treated with ANK soon after NSAIDs and/or corticosteroids failure and those previously treated with other treatment choices. No differences were identified in the DRR of ANK by stratifying patients according with ANK treatment line. Taken together, these findings support that long-term therapeutic outcome is independent of how early ANK treatment is started.

The results emerging from the present study highlight the overall lack of differences in ANK effectiveness based on different time intervals between AOSD onset and ANK introduction as well as according to the different treatment lines. Nevertheless, a faster ANK effectiveness in controlling systemic inflammation and resolving articular manifestations has been identified when patients benefit from IL-1 inhibition as soon after AOSD onset. Differences in articular manifestations and laboratory response tend to settle over time and therapeutic long-term outcome is similar regardless of how early ANK treatment is started. In this perspective, long-term outcome to ANK treatment appears independent of how early IL-1 inhibition is started in AOSD patients. On the other hand, the slower articular response identified in patients treated with ANK in a non-early stage of the disease could explain why articular involvement has been frequently described as less or slowly responsive than other AOSD manifestations. Actually, currently available studies addressing ANK efficacy in AOSD patients deal with a mean delay of 3.5 to 9.3 years from disease onset to the start of ANK treatment (21–23, 36). For these reasons, future studies will have to verify whether this finding reflects a more resistant joint disease in patients failing other treatment strategies or underlies the need for an earlier ANK introduction in order to better manage joint manifestations.

Beyond joint involvement, no other single AOSD manifestations showed significant difference in the rate of resolution at the three follow-up visits, as almost all statistical significances did not overcome Bonferroni correction at the *post-hoc* analysis (Figures 1–3). As regards stratification for different treatment lines, these findings seem to contradict the higher decrease of the systemic score observed among patients treated with ANK as first line treatment approach compared to those previously treated with cDMARDs and other biologics. In this context, no statistically significant differences were observed in the frequency of each clinical manifestation taken individually, while the systemic score seems to highlight a general higher response to ANK administered early.

The main limitation of this study is represented by its retrospective nature. In this regard, initiation of ANK was not made at random and the groups of patients were essentially not comparable at baseline; actually, our experience reflects the real-life world, as more difficult cases probably started ANK earlier, as suggested by the higher systemic score and the higher baseline daily corticosteroid dosages employed when IL-1 inhibition was started earlier. However, baseline differences does not seem to compromise results, as the overall ANK effectiveness did not change between groups despite initial differences, while the frequency of resolution of systemic inflammation resulted faster especially in groups with more severe conditions (higher systemic score and corticosteroid dosage) at the start of ANK. In further support of this, regression analysis did not identify any role for possible baseline confounding factors related to patients' features, AOSD activity and concomitant treatments including the type of AOSD (systemic vs. chronic articular), baseline corticosteroid dosage, the concomitant use of cDMARDs, the systemic score and the severity of joint involvement assessed with the DAS28-CRP, the number of tender joints and the number of swollen joints. Among study limitations, some interesting quantitative variables, including white blood cell count, ferritin serum levels, ESR and CRP, were only available as qualitative data (increased/not increased). Similarly, radiographic progression could not be assessed because of the lack of information.

As no defined criteria are currently applicable for starting or stopping IL-1 inhibition in AOSD patients, the real-life enrollment has allowed statistical analysis including data obtained from subjects treated with ANK in different therapeutic lines and at different moments from AOSD onset. In this regard, the study has been conducted using time cut-offs at 6 and 12 months from disease onset. We could have chosen higher time limits (24, 36 or 48 months since disease onset), but this would not have been helpful in addressing how early IL-1 inhibition should be introduced for treating patients with AOSD.

In conclusion, our results highlight that clinical and therapeutic outcomes are substantially independent of how early ANK treatment is started. However, a faster effectiveness of ANK in controlling systemic inflammation and resolving articular manifestations may be described in patients benefiting from IL-1 inhibition as soon as after AOSD onset. In addition, a lower decrease in the systemic Pouchot score is observed in patients treated with ANK only after the use of cDMARDs and other biologics. These findings could suggest an early introduction of treatment with ANK specifically in patients with articular involvement.

## Data Availability Statement

The datasets generated for this study are available on request to the corresponding author.

## Ethics Statement

The study protocol was approved by the Ethics Committee of the University of Florence (reference number: 364-16OCT2013). Written informed consent was obtained from each patient for the retrospective evaluation of her/his medical chart.

## Author Contributions

AV and LC conceived and designed the study and wrote the first draft of the manuscript. AV performed the statistical analysis. DR revised the final data and the manuscript. All other authors have enrolled patients, collected data and critically reviewed the final draft of the manuscript and approved the submitted version.

### Conflict of Interest

The authors declare that the research was conducted in the absence of any commercial or financial relationships that could be construed as a potential conflict of interest.

## References

[1] Uppal SS Pande IR Kumar A Kailash S Sekharan NG Adya CM. Adult onset Still's disease in northern India: comparison with juvenile onset Still's disease. Br J Rheumatol. (1995) 34:429–34. 7788171 10.1093/rheumatology/34.5.429

[2] Luthi F Zufferey P Hofer MF So AK. “Adolescent-onset Still's disease”: characteristics and outcome in comparison with adult-onset still's disease. Clin Exp Rheumatol. (2002) 20:427–30. 12102485

[3] Hayem F. Is Still's disease an autoinflammatory syndrome? Joint Bone Spine. (2009) 76:7–9. 10.1016/j.jbspin.2008.05.009 19084456

[4] Martini A. Systemic juvenile idiopathic arthritis. Autoimmun Rev. (2012) 12:56–9. 10.1016/j.autrev.2012.07.022 22884552

[5] Rossi-Semerano L Koné-Paut I. Is Still's disease an autoinflammatory syndrome? Int J Inflam. (2012) 2012:480373. 10.1155/2012/480373 22611516 PMC3350968

[6] Rigante D. A systematic approach to autoinflammatory syndromes: a spelling booklet for the beginner. Expert Rev Clin Immunol. (2017) 13:571–97. 10.1080/1744666X.2017.1280396 28064547

[7] Cush JJ Medsger TA Christy WC Herbert DC Cooperstein LA. Adult-onset Still's disease. Clinical course and outcome. Arthritis Rheum. (1987) 30:186–94. 3827959 10.1002/art.1780300209

[8] Pouchot J Sampalis JS Beaudet F Carette S Décary F Salusinsky-Sternbach M. Adult Still's disease: manifestations, disease course, and outcome in 62 patients. Medicine. (1991) 70:118–36. 2005777

[9] Yamaguchi M Ohta A Tsunematsu T Kasukawa R Mizushima Y Kashiwagi H. Preliminary criteria for classification of adult Still's disease. J Rheumatol. (1992) 19:424–30. 1578458

[10] Fautrel B Zing E Golmard JL Le Moel G Bissery A Rioux C. Proposal for a new set of classification criteria for adult-onset Still disease. Medicine. (2002) 81:194–200. 10.1097/00005792-200205000-00003 11997716

[11] Rau M Schiller M Krienke S Heyder P Lorenz H Blank N Clinical manifestations but not cytokine profiles differentiate adult-onset Still's disease and sepsis. J Rheumatol. (2010) 37:2369–76. 10.3899/jrheum.100247 20810496

[12] Ruscitti P Cipriani P Masedu F Iacono D Ciccia F Liakouli V. Adult-onset Still's disease: evaluation of prognostic tools and validation of the systemic score by analysis of 100 cases from three centers. BMC Med. (2014) 14:194. 10.1186/s12916-016-0738-8 27903264 PMC5131497

[13] Iliou C Papagoras C Tsifetaki N Voulgari PV Drosos AA. Adult-onset Still's disease: clinical, serological and therapeutic considerations. Clin Exp Rheumatol. (2013) 31:47–52. 23010097

[14] Papagoras C Chrysanthopoulou A Mitsios A Arampatzioglou A Ritis K Skendros P. Autophagy inhibition in adult-onset Still's disease: still more space for hydroxychloroquine? Clin Exp Rheumatol. (2017) 35:133–4. 29148405

[15] Horneff G Schmeling H Biedermann T Foeldvari I Ganser G Girschick HJ. The German etanercept registry for treatment of juvenile idiopathic arthritis. Ann Rheum Dis. (2004) 63:1638–44. 10.1136/ard.2003.014886 15115709 PMC1754849

[16] Grom AA. Natural killer cell dysfunction: a common pathway in systemic-onset juvenile rheumatoid arthritis, macrophage activation syndrome, and hemophagocytic lymphohistiocytosis? Arthritis Rheum. (2004) 50:689–98. 10.1002/art.20198 15022306

[17] Ramanan AV Grom AA. Does systemic-onset juvenile idiopathic arthritis belong under juvenile idiopathic arthritis? Rheumatology. (2005) 44:1350–3. 10.1093/rheumatology/keh710 15956091

[18] Frosch M Roth J. New insights in systemic juvenile idiopathic arthritis: from pathophysiology to treatment. Rheumatology. (2008) 47:121–5. 10.1093/rheumatology/kem271 17971384

[19] Sota J Insalaco A Cimaz R Alessio M Cattalini M Gallizzi R. Drug retention rate and predictive factors of drug survival for interleukin-1 inhibitors in systemic juvenile idiopathic arthritis. Front Pharmacol. (2019) 9:1526. 10.3389/fphar.2018.01526 30670972 PMC6331484

[20] Nordström D Knight A Luukkainen R van Vollenhoven R Rantalaiho V Kajalainen A. Beneficial effect of interleukin 1 inhibition with anakinra in adult-onset Still's disease. An open randomized multicenter study. J Rheumatol. (2008) 39:2008–11. 10.3899/jrheum.111549 22859346

[21] Giampietro C Ridene M Lequerre T Costedoat Chalumeau N Amoura Z Sellam J. Anakinra in adult-onset Still's disease: long-term treatment in patients resistant to conventional therapy. Arthritis Care Res. (2013) 65:822–6. 10.1002/acr.21901 23225779

[22] Ortiz-Sanjuán F Blanco R Riancho-Zarrabeitia L Castañeda S Olivé A Riveros A. Efficacy of anakinra in refractory adult-onset Still's disease: multicenter study of 41 patients and literature review. Medicine. (2015) 94:e1554. 10.1097/MD.0000000000001554 26426623 PMC4616841

[23] Cavalli G Franchini S Aiello P Guglielmi B Berti A Campochiaro C. Efficacy and safety of biological agents in adult-onset Still's disease. Scand J Rheumatol. (2015) 44:309–14. 10.3109/03009742.2014.992949 25656459

[24] Colafrancesco S Priori R Valesini G Argolini L Baldissera E Bartoloni E. Response to interleukin-1 inhibitors in 140 Italian patients with adult-onset Still's disease: a multicentre retrospective observational study. Front Pharmacol. (2017) 8:369. 10.3389/fphar.2017.00369 28659802 PMC5469286

[25] Vastert SJ de Jager W Noordman BJ Holzinger D Kuis W Prakken BJ. Effectiveness of first-line treatment with recombinant interleukin-1 receptor antagonist in steroid-naive patients with new-onset systemic juvenile idiopathic arthritis: results of a prospective cohort study. Arthritis Rheumatol. (2014) 66:1034–43. 10.1002/art.38296 24757154

[26] Nigrovic PA Mannion M Prince FH Zeft A Rabinovich CE van Rossum MA. Anakinra as first-line disease-modifying therapy in systemic juvenile idiopathic arthritis: report of forty-six patients from an international multicenter series. Arthritis Rheum. (2011) 63:545–55. 10.1002/art.30128 21280009

[27] Saccomanno B Tibaldi J Minoia F Bagnasco F Pistorio A Guariento A. Predictors of effectiveness of anakinra in systemic juvenile idiopathic arthritis. J Rheumatol. (2019) 46:416–21. 10.3899/jrheum.180331 30647180

[28] Ter Haar NM van Dijkhuizen EHP Swart JF van Royen-Kerkhof A El Idrissi A Leek AP Treat-to-target using first-line recombinant interleukin-1 receptor antagonist monotherapy in new-onset systemic juvenile idiopathic arthritis: results from a five year follow-up study. Arthritis Rheumatol. (2019) 71:1163–73. 10.1002/art.40865 30848528 PMC6617757

[29] Vitale A Cavalli G Colafrancesco S Priori R Valesini G Argolini LM. Long-term retention rate of anakinra in adult onset Still's disease and predictive factors for treatment response. Front Pharmacol. (2019) 10:296. 10.3389/fphar.2019.00296 31001115 PMC6454864

[30] Fransen J Creemers MC Van Riel PL. Remission in rheumatoid arthritis: agreement of the disease activity score (DAS28) with the ARA preliminary remission criteria. Rheumatology. (2004) 43:1252–5. 10.1093/rheumatology/keh297 15238643

[31] Wells GA Boers M Shea B Brooks PM Simon LS Strand CV. Minimal disease activity for rheumatoid arthritis: a preliminary definition. J Rheumatol. (2005) 32:2016–24. 16206362

[32] Cantarini L Lucherini OM Frediani B Brizi MG Bartolomei B Cimaz R. Bridging the gap between the clinician and the patient with cryopyrin-associated periodic syndromes. Int J Immunopathol Pharmacol. (2011) 24:827–36. 10.1177/039463201102400402 22230390

[33] Rossi-Semerano L Fautrel B Wendling D Hachulla E Galeotti C Semerano L. Tolerance and efficacy of off-label anti-interleukin-1 treatments in France: a nationwide survey. Orphanet J Rare Dis. (2015) 10:19. 10.1186/s13023-015-0228-7 25758134 PMC4340831

[34] Maria AT Le Quellec A Jorgensen C Touitou I Riviere S Guilpain P. Adult onset Still's disease (AOSD) in the era of biologic therapies: dichotomous view for cytokine and clinical expressions. Autoimmun Rev. (2014) 13:1149–59. 10.1016/j.autrev.2014.08.032 25183244

[35] Moulis G Sailler L Astudillo L Pugnet G Arlet P. May anakinra be used earlier in adult onset Still disease? Clin Rheumatol. (2010) 29:1199–200. 10.1007/s10067-010-1459-6 20428907

[36] Lequerré T Quartier P Rosellini D Alaoui F De Bandt M Mejjad O. Interleukin-1 receptor antagonist (anakinra) treatment in patients with systemic-onset juvenile idiopathic arthritis or adult onset Still disease: preliminary experience in France. Ann Rheum Dis. (2008) 67:302–8. 10.1136/ard.2007.076034 17947302

