# Supplementary material for: Comparison of Early vs. Delayed Anakinra Treatment in Patients With Adult Onset Still's Disease and Effect on Clinical and Laboratory Outcomes
Source: Front Med (Lausanne). 2020 Feb 21;7:42. doi: 10.3389/fmed.2020.00042 (PMC7047849; doi:10.3389/fmed.2020.00042)
Supplement: Supplementary file 1 [file Table_1.DOCX]

**Supplementary table 1**: details about the frequency of effectiveness to anakinra, primary and secondary inefficacy in the different subgroups of patients identified in the study. Abbreviations: ANK, anakinra; cDMARDs, conventional disease modifying anti-rheumatic drugs. Values are patient numbers and, in brackets, percentages referring to each patient group.

|  | **Effectiveness, n (%)** | **Primary inefficacy** | **Secondary inefficacy** |
| --- | --- | --- | --- |
| Group <6 months (n=40) | 35 (87.5) | 2 (5) | 3 (7.5) |
| Group >6 months (n=101) | 82 (81.2) | 13 (12.9) | 6 (5.9) |
| Group <12 months (n=65) | 56 (86.2) | 5 (7.7) | 4 (6.2) |
| Group >12 months (n=76) | 61 (80.3) | 10 (13.2) | 5 (6.6) |
| ANK first line (n=19) | 18 (94.7) | 0 (0.0) | 1 (5.3) |
| ANK preceded only by cDMARDs (n=93) | 77 (82.8) | 10 (10.6) | 6 (6.6) |
| ANK preceded by cDMARDs and other biologics (n=29) | 23 (79.3) | 4 (13.8) | 2 (6.9) |
