# Supplementary material for: Comparison of Early vs. Delayed Anakinra Treatment in Patients With Adult Onset Still's Disease and Effect on Clinical and Laboratory Outcomes
Source: Front Med (Lausanne). 2020 Feb 21;7:42. doi: 10.3389/fmed.2020.00042 (PMC7047849; doi:10.3389/fmed.2020.00042)
Supplement: Supplementary file 2 [file Table_2.DOCX]

**Supplementary table 2:** systemic score according to Rau et al 2010 at the start of treatment (baseline) and at 3-, 6- and 12-month assessments in different subgroups of patients identified in the study. The mean decreases in systemic score after 3, 6 and 12 months from the start of anakinra are also reported. Values are provided as mean±standard deviation. Abbreviations: ANK, anakinra; cDMARDs, conventional disease modifying anti-rheumatic drugs; n: number of patients for each subgroup.

|  | **Baseline systemic score** | ***p*-value** | **Decrease in systemic score after 3 months** | ***p*-value** | **Systemic score at 3-month assessment** | ***p*-value** | **Decrease in systemic score after 6 months** | ***p*-value** | **Systemic score at 6-month assessment** | ***p*-value** | **Decrease in systemic score after 12 months** | ***p*-value** | **Systemic score at 12-month assessment** | ***p*-value** |
| --- | --- | --- | --- | --- | --- | --- | --- | --- | --- | --- | --- | --- | --- | --- |
| **Group <6 months (n=40)** | 6.3±1.8 | 0.006 | 5.6±1.4 | 0.006 | 0.96±1.0 | 0.67 | 5.9±1.2 | <0.001 | 0.64±0.99 | 0.02 | 5.8±1.1 | 0.001 | 0.47±1.07 | 0.89 |
| **Group >6 months (n=101)** | 5.23±1.9 |  | 4.1±2.1 |  | 0.96±1.4 |  | 4.5±2.1 |  | 0.52±1.19 |  | 4.7±2.4 |  | 0.63±1.21 |  |
| **Group <12 months (n=65)** | 6.2±1.8 | <0.001 | 4.9±2.5 | 0.012 | 1.00±1.06 | 0.78 | 5.1±2.5 | 0.001 | 0.67±1.1 | 0.22 | 5.1±2.4 | 0.002 | 0.57±1.2 | 0.37 |
| **Group >12 months (n=76)** | 5.0±1.8 |  | 3.6±2.2 |  | 0.93±1.23 |  | 3.9±2.3 |  | 0.53±1.01 |  | 3.9±2.5 |  | 0.48±1.04 |  |
| **ANK first line (n=19)** | 5.8±1.4 | 0.17 | 5.3±2.0 | 0.01^A^ | 0.47±0.74 | 0.047* | 5.4±1.5 | 0.02^A^ | 0.2±0.4 | 0.11 | 5.7±1.2 | 0.04^A^ | 0.13±0.4 | 0.31 |
| **ANK preceded only by cDMARDs (n=93)** | 5.5±1.9 |  | 4.6±2.0 |  | 1.00±1.15 |  | 4.9±2.2 |  | 0.7±1.1 |  | 4.9±2.5 |  | 0.63±1.3 |  |
| **ANK preceded by cDMARDs and other biologics (n=29)** | 5.2±1.0 |  | 3.3±2.1 |  | 1.3±1.3 |  | 4.5±1.0 |  | 0.8±1.1 |  | 5.0±1.2 |  | 0.57±0.6 |  |

Significances at the post-hoc analysis when multiple comparisons were performed: A= “*ANK first line*” group *versus* “*ANK preceded by cDMARDs and other biologics*” group. The sign “*” indicates a lack of significance at the Bonferroni correction (*p*>0.017).
