# Supplementary material for: Comparison of Early vs. Delayed Anakinra Treatment in Patients With Adult Onset Still's Disease and Effect on Clinical and Laboratory Outcomes
Source: Front Med (Lausanne). 2020 Feb 21;7:42. doi: 10.3389/fmed.2020.00042 (PMC7047849; doi:10.3389/fmed.2020.00042)
Supplement: Supplementary file 3 [file Table_3.DOCX]

**Supplementary table 3:** mean daily corticosteroid dosage and mean decrease in the corticosteroid dosage at 3-, 6- and 12-month assessments compared to the start of anakinra in the different subgroups of patients identified in the study. Values provided are reported as mg/day of prednisone or equivalent molecule (mean±standard deviation). Abbreviations: ANK, anakinra; cDMARDs, conventional disease modifying anti-rheumatic drugs; CS, corticosteroids; n: number of patients for each subgroup.

Significances at the post-hoc analysis: A= “*ANK first line*” group versus “*ANK preceded by cDMARDs and other biologics*” group; B= “*ANK preceded by cDMARDs and other biologics” versus* “*ANK preceded only by cDMARDs*” group; C= “*ANK first line*” group *versus* “*ANK preceded only by cDMARDs*” group.

|  | **Baseline CS dosage** | ***p*-value** | **Decrease in CS dosage after 3 months** | ***p*-value** | **CS dosage at 3-month visit** | ***p*-value** | **Decrease in CS dosage after 6 months** | ***p*-value** | **CS dosage at 6-month visit** | ***p*-value** | **Decrease in CS dosage after 12 months** | ***p*-value** | **CS dosage at 12-month visit** | ***p*-value** |
| --- | --- | --- | --- | --- | --- | --- | --- | --- | --- | --- | --- | --- | --- | --- |
| **Group <6 months (n=40)** | 27.8±18.9 | <0.0001 | 16.9±18.5 | 0.064 | 14.1±12.5 | 0.27 | 23.8±18.2 | 0.002 | 8.4±5.5 | 0.37 | 26.7±15.8 | 0.011 | 5.5±2.6 | 0.33 |
| **Group >6 months (n=101)** | 15.8±17.2 |  | 10.4±14.9 |  | 11.9±10.5 |  | 10.4±14.9 |  | 8.5±7.2 |  | 12.4±14.9 |  | 6.4±5.1 |  |
| **Group <12 months (n=65)** | 25.5±20.7 | 0.001 | 13.5±20.0 | 0.001 | 12.4±12.0 | 0.94 | 18.5±21.0 | 0.007 | 7.2±4.8 | 0.56 | 18.9±21.3 | 0.032 | 6.2±5.4 | 0.54 |
| **Group >12 months (n=76)** | 14.2±14.8 |  | 5.0±12.5 |  | 12.1±9.7 |  | 7±2.9 |  | 9.8±8.0 |  | 9.7±14.4 |  | 6.6±4.2 |  |
| **ANK first line (n=19)** | 32.9±14.4 | 0.001^A,B^ | 22.1±11.0 | <0.0001^A,C^ | 10.6±7.2 | 0.60 | 29.2±14.3 | <0.0001^A,B^ | 6.1±1.4 | 0.18 | 31.1±14. | 0.004^A^ | 5.3±0.6 | 0.11 |
| **ANK preceded only by cDMARDs (n=93)** | 16.8±15.1 |  | 7.9±15.8 |  | 12.7±11.8 |  | 11.7±16.6 |  | 8.1±7.3 |  | 13.8±15.9 |  | 5.6±4.3 |  |
| **ANK preceded by cDMARDs and other biologics (n=29)** | 8.1±3.8 |  | 0.6±1.2 |  | 12.3±9.7 |  | 3.3±4.7 |  | 10.3±6.0 |  | 5.1±4.52 |  | 8.0±5.8 |  |
