# Supplementary material for: Comparison of Early vs. Delayed Anakinra Treatment in Patients With Adult Onset Still's Disease and Effect on Clinical and Laboratory Outcomes
Source: Front Med (Lausanne). 2020 Feb 21;7:42. doi: 10.3389/fmed.2020.00042 (PMC7047849; doi:10.3389/fmed.2020.00042)
Supplement: Supplementary file 4 [file Table_4.DOCX]

|  | **Disease duration at the start of ANK** | **Systemic score** | **Steroid dosage (mg/day)** | **Concomitant use of cDMARDs** | **DAS28-CRP** | **Number of tender joints** | **Number of swollen joints** | **Age at onset** | **Sex** | **Type of AOSD** |
| --- | --- | --- | --- | --- | --- | --- | --- | --- | --- | --- |
| **Effectiveness at 6 months** | 0.276 | 0.132 | 0.630 | 0.375 | 0.051 | 0.341 | 0.817 | 0.321 | 0.890 | 0.729 |
| **Effectiveness at 12 months** | 0.395 | 0.445 | 0.606 | 0.767 | 0.204 | 0.208 | 0.440 | 0.241 | 0.900 | 0.194 |
| **Systemic score of zero at 6 months** | 0.886 | 0.732 | 0.450 | 0.491 | 0.663 | 0.349 | 0.609 | 0.232 | 0.836 | 0.163 |
| **Systemic score of zero at 12 months** | 0.391 | 0.477 | 0.828 | 0.480 | 0.264 | 0.783 | 0.062 | 0.543 | 0.160 | 0.133 |
| **DAS28-CRP<2.6 at 6 month** | 0.662 | 0.648 | 0.635 | 0.774 | 0.920 | 0.359 | 0.529 | 0.634 | 0.376 | 0.430 |
| **DAS28-CRP<2.6 at 12 month** | 0.590 | 0.548 | 0.088 | 0.075 | 0.817 | 0.795 | 0.671 | 0.487 | 0.912 | 0.976 |
| **Inflammatory markers resolved at 6 month** | 0.725 | 0.220 | 0.513 | 0.356 | 0.052 | 0.178 | 0.302 | 0.182 | 0.060 | 0.307 |
| **Inflammatory markers resolved at 6 month** | 0.987 | 0.850 | 0.986 | 0.824 | 0.984 | 0.984 | 0.959 | 0.882 | 0.978 | 0.960 |

**Supplementary table 4**. *P*-values obtained at binary stepwise regression analysis; in the first row independent variables collected at the start of anakinra have been listed, while dependent variables have been reported in the first column. Abbreviations: ANK, anakinra cDMARDs, conventional disease modifying anti-rheumatic drugs, DAS28-CRP: disease activity score in 28 Joints-C-reactive protein.
